# Preventing tuberculosis in paediatric kidney transplant recipients: is there a role for BCG immunisation pre-transplantation in low tuberculosis incidence countries?

**DOI:** 10.1007/s00467-020-04844-5

**Published:** 2020-11-27

**Authors:** Alasdair Bamford, Garth Dixon, Nigel Klein, Stephen D. Marks, Nicole Ritz, Steven B. Welch, Marc Tebruegge

**Affiliations:** 1 Department of Paediatric Infectious Diseases, Great Ormond Street Hospital for Children NHS Foundation Trust, London, UK; 2 University College London Great Ormond Street Institute of Child Health, NIHR Great Ormond Street Hospital Biomedical Research Centre, London, UK; 3 Department of Paediatirc Microbiology, Great Ormond Street Hospital for Children NHS Foundation Trust, London, UK; 4 Department of Paediatric Nephrology, Great Ormond Street Hospital for Children NHS Foundation Trust, London, UK; 5 University of Basel Children’s Hospital, Paediatric Infectious Disease and Vaccinology Department, Migrant Health Service, Basel, Switzerland; 6 Department of Paediatrics, Royal Children’s Hospital Melbourne, University of Melbourne, Melbourne, Australia; 7 Birmingham Chest Clinic and Heartlands Hospital, University Hospitals Birmingham, Birmingham, UK; 8 Department of Paediatric Infectious Diseases & Immunology, Evelina London Children’s Hospital, Guy’s and St. Thomas’ NHS Foundation Trust, London, UK

**Keywords:** Tuberculosis, BCG, Chronic kidney disease, Transplant, Children

## Abstract

The risk of tuberculosis (TB) disease is increased in children with chronic kidney disease (CKD), even higher in stage 5 CKD/kidney failure and especially high after kidney transplantation due to immunosuppression. TB disease may follow recent primary infection, or result from reactivation of latent infection. Reactivation is more common in adults, while progression following primary infection makes up a greater proportion of disease in children. Recommendations for preventing TB disease in some low TB incidence countries have previously included offering Bacillus Calmette-Guérin (BCG) vaccine to all children listed for kidney transplant if they had not received this as part of previous national immunisation programmes. Based on the available evidence, we recommend modifying this practice, focusing instead on awareness of risk factors for TB exposure, infection and disease and the use of appropriate testing strategies to identify and treat TB infection and disease.

**Supplementary Information:**

The online version contains supplementary material available at 10.1007/s00467-020-04844-5.

## Summary

### Diagnosing tuberculosis infection

Assessment for tuberculosis (TB) infection and disease has three components:

- Assessment of risk factors for TB exposure, or confirmed history of TB exposure
- Diagnostic immunological tests for TB infection (tuberculin skin test (TST) and interferon-gamma release assays (IGRAs)), which are less sensitive in the presence of immunosuppression and potentially also in chronic kidney disease (CKD)
- Clinical assessment for symptoms of TB disease followed by appropriate microbiological, histological and radiological investigations as indicated.

### Bacillus Calmette-Guérin vaccine

Bacillus Calmette-Guérin (BCG) is a live-attenuated vaccine which remains viable for several years after administration. BCG has a risk of local side effects, and more importantly of disseminated BCG disease when immunosuppression for transplantation is commenced. Practice in low incidence countries in Europe varies between universal, risk-based and no BCG immunisation. Risk-based immunisation is usually based on the risk of TB exposure, for example if there is travel to or parental origin from countries with high TB incidence. In some countries, BCG is also recommended based on increased host susceptibility, irrespective of the exposure risk.

### Current practice in low TB incidence countries

Practices relating to screening for TB and administration of BCG to children with chronic kidney disease (CKD) vary between countries. As an example, in paediatric kidney transplant centres in the UK, practice has previously been to:

- Test all children listed for kidney transplant for TB infection using immunological tests.
- If TB tests suggest infection, they are assessed for TB disease and if this is confirmed they are treated for TB disease.
- If there is no evidence of TB disease, children with positive immunological tests are offered treatment for latent TB infection (LTBI).
- If tests for TB infection are negative they are offered immunisation with BCG if not previously BCG immunised.

### Recommended updated practice for low TB incidence settings:

- Thoroughly screen children with CKD for TB infection ideally prior to any immunosuppression and/or deceased or living donor kidney transplantation, and treat LTBI if the screening tests are positive and TB disease has been excluded.
- CKD and planning for kidney transplantation alone are not indications for BCG immunisation. BCG should be given according to the current risk factor-based indications as listed in current national guidance, but is contraindicated in the context of immunosuppressive therapy for kidney transplantation.
- If there is a risk or history of significant TB exposure (born in or travel to high risk area, or confirmed exposure) but tests for TB infection are negative, discuss the need for possible treatment for LTBI with a TB specialist. This is because these tests may have lower sensitivity in kidney disease, especially if performed after immunosuppression has already commenced.
- Once immunosuppression has been started there should be vigilance for symptoms of possible TB disease, and regular screening by history for any new possible exposures to TB.

### Treatment of TB infection, BCG and TB disease

Treatment of TB infection or disease, or of disseminated BCG should involve paediatric infectious diseases and nephrology teams, with input from a specialist pharmacist. The most commonly used drugs used for treating TB infection and disseminated BCG (rifampicin and isoniazid) do not require dose adjustment in CKD or after transplantation, but may do during dialysis. Both drugs also have the potential for interaction with other medications used in CKD or for immune suppression, and may have additive toxicities. Use of more complex regimens for TB disease, including for drug-resistant disease, should all be assessed for potential need for dose adjustments and for interactions and toxicities.

## Review

### Epidemiology of paediatric chronic kidney disease

The reported prevalence of Stage III, IV and V (GFR < 60) CKD with estimated glomerular filtration rate less than 60 ml/min/1.73 m^2^ in children and young people aged less than 20 years in Europe is 55 to 60 per million of age-related population (incidence 11–12 per million of age-related population); the global estimate is a prevalence of 65 per million of age-related population and incidence of 9 per million of age-related population [1]. These figures, especially the global estimates, are likely to be significant underestimates as there are only limited or no data available from some low-income regions. CKD is frequently asymptomatic and reporting practices vary greatly between countries, with more detailed information available in higher income settings. A recent survey and review of national paediatric kidney replacement therapy registries highlighted variation in practice and significant gaps in coverage [2].

The incidence of CKD in children less than 16 years old in the UK was 9 per million of age-related population in 2016. A higher prevalence was noted in children from ethnic minorities, especially South Asian children in whom the prevalence was almost double the whole population estimate [3]. This observed difference is thought to be in part attributable to ethnic differences in rates of inherited kidney disease [4].

### Epidemiology of paediatric TB

TB infection can be divided into that causing disease (TB disease or active TB) and that not causing disease (latent TB infection (LTBI)), although there is increasing evidence that these are not discrete entities, but rather different states along a continuum [5]. Estimating the burden of TB disease in children is challenging, in part due to under-recognition, difficulties in establishing a diagnosis and incomplete reporting in countries with some of the highest burden of disease [6]. Nevertheless, it is estimated that in 2017, 1 million children globally developed TB disease, while the number of TB-related deaths was approximately 150,000. The majority of these cases occurred in the African, South-East Asian and Western Pacific regions [7]. The reported rate of TB disease in children less than 15 years old in England born outside the UK was 7.3 per 100,000 in 2017, while the rate for children born in the UK was 1.4 per 100,000. When combining reports from adults and children, rates were highest for individuals of non-white ethnicity born outside the UK [8].

The global prevalence of LTBI is much greater than that of TB disease. Approximately 6% of children under 15 years of age were estimated to be infected with TB in 2016, equating to around 90 million children worldwide [9].

### TB in chronic kidney disease, kidney replacement therapy and kidney transplantation

Most data on TB in CKD, kidney replacement therapy and kidney transplantation come from studies in adults, with only a few case series reported in children. The reasons for higher observed rates of TB disease in kidney patients are likely to be multifactorial, relating to immune impairment secondary to CKD itself, underlying causative disease, co-morbidity, immunosuppressive therapy and socio-economic factors and common risks for kidney disease and exposure to TB [10].

A review completed for the 2011 version of the UK NICE TB guidelines estimated the relative risk of TB disease in adults was 10 to 25 for CKD or haemodialysis patients and 37 for kidney transplant recipients, although it should be taken into account that this was based on data from more than 20 years ago when the incidence of TB in the UK was significantly higher. A more recent report in adults from the UK found that the cumulative incidence of TB disease was 1267 per 100,000 population in haemodialysis patients (95% confidence interval (CI): 630–1904; 85 times higher than the background UK TB rate), 398 per 100,000 in patients on peritoneal dialysis (95% CI: 80–1160; 26 times higher than the background UK rate); and 522 per 100,000 in kidney transplant recipients (CI: 137–909; 35 times higher than the background UK rate) [11].

Overall, the risk of TB disease in adult solid organ transplant patients is estimated to be 20 to 74 times higher than in the general population [12]. This is largely attributable to immunosuppression. Extrapulmonary and disseminated TB are more common in this patient group. Symptoms of disease are more likely to be non-specific and TB-related mortality is much higher than in the general population [13].

Data on TB in paediatric CKD and kidney transplant recipients are scarce. Risk varies depending on background TB incidence in the general population. Rates of TB disease following kidney transplantation in children have been reported to be between 8 and 9.7% in highly TB endemic areas [13]. In a South African case series, 7 of 72 (9.7%) children undergoing kidney transplantation developed TB disease [14]. In contrast, in a Spanish retrospective study covering a 26-year period, only 3 of 345 (0.9%) children undergoing kidney transplantation were subsequently diagnosed with TB disease, although this rate was still much higher than that observed in the general population of a similar age [15].

TB disease in children is more commonly due to progression after primary infection rather than reactivation of latent infection; immunosuppression after organ transplantation increases both the likelihood of progression after infection, and the risk of reactivation of LTBI. Children also have higher rates of disseminated and extrapulmonary TB disease than adults, as well as higher TB-related mortality [16].

### Screening for LTBI and TB disease in CKD patients before and after kidney transplant

In view of the increased risk of TB disease in children with CKD, thorough assessment for LTBI and TB disease is essential, especially for those receiving kidney replacement therapy or proceeding to kidney transplantation. TB infection is diagnosed by demonstrating immunological reaction to *Mycobacterium tuberculosis* antigens, using either a TST or an IGRA.

The TST demonstrates a type IV hypersensitivity reaction to intradermally injected purified protein derivative, a heterogenous mixture of approximately 200 mycobacterial peptide antigens. The TST has several limitations, including the need for the patient to return for the reading (i.e. requiring two visits), and a degree of subjectivity with substantial inter-operator variability when reading the test result. Importantly, there is strong evidence to suggest that the TST has reduced sensitivity in individuals with primary or secondary immunodeficiency, including patients receiving immunosuppressive medication. The TST is also not specific for TB infection, and positive results can be caused by BCG immunisation or infection with non-tuberculous mycobacteria (NTM) [17].

Currently, there are two commonly available commercial IGRAs, the QuantiFERON-TB (QFT) Gold Plus and the T-SPOT.TB assay. Both tests are functional assays that rely on measuring interferon-gamma produced in response to stimulation with only two relatively *M. tuberculosis*–specific peptides (ESAT-6 and CFP-10). IGRAs are more specific than the TST, as those peptides are absent in BCG; prior BCG immunisation does therefore not impact on test results. However, a few NTM species do express those peptides, including *M. marinum*, *M. kansasii* and *M. szulgai*, and therefore false-positive results can occur in patients infected with NTM [18, 19]. IGRAs have relatively poor reproducibility when serial testing is performed [20], and considerable variability associated with delays in incubation and variations in environmental temperatures has been reported [21, 22]. There are substantial data showing that IGRAs perform less well, and are more likely to give indeterminate results, in young children than in adults [23–25].

It is important to consider the reliability of IGRAs in the presence of CKD when interpreting test results. There is strong evidence that the performance of IGRAs is impaired in the context of immunodeficiency (e.g. HIV-infected patients with low CD4^+^ T cell counts) [25–27]. Data on the performance of IGRAs in patients with CKD are conflicting [28–31]. A recent study reported a high degree of discordance between QFT and T-SPOT. TB and TST results in kidney dialysis patients who had been exposed to a healthcare worker with infectious TB, indicating that all three immune-based TB tests may have suboptimal performance in this patient population [32].

In accordance with guidance from the European Centre for Disease Prevention and Control (ECDC) and the Tuberculosis Network European Trials Group (TBNET) [13, 33], the authors of this review recommend that both TST and IGRA are performed in parallel, in individuals with impaired immune function, including children with CKD, when investigating for TB infection, as chronic uraemia impairs innate as well as adaptive T cell–mediated immune responses [34]. If either test produces a positive result, the presence of TB infection should be assumed and LTBI treatment should be initiated once TB disease has been excluded. It should be noted that BCG is contraindicated in children with a history of TB infection. Further guidance on the management of TB infection in the pre-transplantation setting can be found in the TBNET consensus statement [13]. Children with significant CKD in a low TB incidence country and significant confirmed TB contact should be considered for LTBI treatment even if TST and IGRA results are negative in view of the likely lower sensitivity of these tests in the context of severe kidney impairment and/or immunosuppression. The significance of possible TB exposure should be assessed in conjunction with a paediatric TB specialist.

In the post-transplantation setting, once immunosuppressive medication has been initiated, both TST and IGRAs should be regarded as unreliable for the same reasons as discussed above. With regards to IGRAs, clinical studies have produced conflicting results, with some authors stipulating that their performance is sustained, and others concluding that their performance is impaired [13, 30, 35]. Some studies have been limited by the absence of a true gold standard test for LTBI, which complicates their interpretation. More recent data from in vitro models show that a range of immunosuppressive agents, including corticosteroids, anti-TNF-alpha agents and calcineurin inhibitors have detrimental effects on the performance of IGRAs [36–38]. Therefore, negative TST and IGRA results post-transplantation must be interpreted with great caution. A positive test result remains useful, but currently it is impossible to determine whether a negative result indicates absence of LTBI or alternatively a false-negative test result due to the underlying medical condition and/or immunosuppressive therapy.

### Bacillus Calmette-Guérin vaccine

#### BCG development and immunisation policies

Bacillus Calmette-Guérin (BCG) is an attenuated strain of *Mycobacterium bovis* used as a live vaccine against TB. BCG has been included in the WHO Expanded Programme on Immunisation since 1974.

Most vaccines included in universal vaccination programmes are recommended in patients with CKD. BCG is included in the national schedule for some countries for all children and in other countries, only in children at risk of TB infection. In some countries, CKD is considered a risk factor for TB disease in itself and it is therefore recommended for all these children if not previously administered [39–41]. A recent survey in 18 tertiary paediatric nephrology centres from 12 European countries showed that BCG was recommended as part of the national schedule in five countries, recommended in CKD in three countries and had no specific recommendation in the remaining four countries [39].

#### BCG efficacy

Vaccination with BCG has been shown to provide substantial protection against tuberculous meningitis and disseminated TB in the general population. A systematic review identified six randomised controlled trials (RCTs) that reported on the protective efficacy of BCG against meningeal and miliary TB, which included 157,264 participants [42]. Vaccine protection was found to be substantial (RR 0.15, 95% CI 0.08–0.31), reducing severe TB in vaccinated individuals by 85%. Protection was highest when the vaccination was performed in the neonatal period, with 90% reduction of severe TB (RR 0.10, 95% CI 0.01–0.77), and in school-age children who were TST negative, with 92% reduction of severe disease.

Protective efficacy against pulmonary TB was also estimated in the same meta-analysis, including 309,300 participants from 18 studies [42]. Vaccine protection against pulmonary TB varied considerably in different subgroups depending on study design, age of vaccination, TST positivity and geographical location (distance from the equator). Analysis in the different subgroups showed the efficacy was higher in studies with lower risk of diagnostic detection bias (60%, 95% CI 36–75%). Among those vaccinated as neonates, the relative risk (RR) for pulmonary TB was 0.40 (95% CI 0.28-0.56). Among school-age children who were TST negative at the time of vaccination, protective efficacy was higher (RR 0.25, 95% CI 0.21–0.31); however, protection was low among older age groups who were TST negative (RR 0.88, 95% CI 0.58–1.31). BCG also protects against LTBI as shown in a systematic review including 3855 participants from 14 studies [43]. Studies specifically investigating vaccine efficacy in special patient subsets, such as patients with CKD, are lacking.

#### BCG risk

Complications following vaccination with the live-attenuated BCG strain are rare, but well described [44]. These include regional and extra-regional localised disease, such as BCG abscess and axillary lymphadenitis, which generally have a good prognosis [45], but may potentially delay proceeding to kidney transplantation. The most severe complication is disseminated BCG disease, which in the vast majority of cases is associated with an underlying immunodeficiency, usually primary (severe combined immunodeficiency, chronic granulomatous disease and other cell-mediated immune defects) or HIV infection [46, 47]. Mortality in such cases is very high, with fatal outcome in up to 80%, although directly attributable mortality is difficult to determine, due to the high frequency of other co-morbidities and the presence of other opportunistic infections [48].

BCG immunisation for children with CKD or undergoing kidney transplantation has previously been advocated in some low incidence settings [39]. However, some caution for the use has been expressed, in particular for those children who are receiving immunosuppressive therapy or are known to have an underlying immunodeficiency. Systematic data on the safety of BCG immunisation in children with CKD are lacking, but equally no case reports have been published on severe adverse effects of BCG immunisations in this setting. In the absence of safety data on BCG immunisation in patients with CKD or kidney transplant recipients, observations from liver transplant patients may help to guide decision making. In a case series from Japan no patient was diagnosed with BCG disease among 144 patients who received the BCG vaccine within 4 weeks to 4 years before liver transplantation [49]. In addition, five patients were identified who received BCG less than 4 weeks before or after liver transplantation and these also experienced no adverse events in relation to BCG immunisation.

One case of disseminated BCG disease has been reported in an adult kidney transplant patient, who had previously received intravesical BCG therapy for uroepithelial carcinoma [50]. Despite the lack of data on the risk of BCG-related complications post solid organ transplant, it is widely accepted that there is a potential risk from any live vaccine used, including BCG [51, 52].

#### Treatment of BCG-related disease

If BCG disease occurs, treatment will depend on severity and whether disease is localised or disseminated. Other factors to be considered include the degree of immunosuppression, as well as the causative BCG strain, as the antibiotic susceptibility pattern varies between strains [53]. Treatment for disseminated disease should always be supervised by a specialist TB paediatrician. Commonly used drugs included isoniazid and rifampicin. Isoniazid is potentially hepatotoxic and may cause pyridoxine-dependent peripheral neuropathy if the child is nutritionally compromised; rifampicin is a potent cytochrome P450 enzyme inducer leading to numerous potential drug-drug interactions. Although for both drugs, many clinicians do not recommend dose adjustments in CKD, they may need adjustments during peritoneal or haemodialysis [54].

#### Summary

In summary, there is very limited evidence for efficacy of BCG in children with CKD on kidney replacement therapy or prior to kidney transplantation. There are potential risks to administration of BCG and the treatment of BCG complications, if they occur in the context of kidney transplantation. Kidney replacement therapy and pre-transplantation assessment alone are not indications for BCG in low TB incidence settings. The authors propose that BCG vaccine should only be given in the presence of additional risk factors for TB exposure as per relevant national immunisation guidance.

### Treatment of LTBI and TB disease in children with chronic kidney disease

#### Latent TB infection

No data exist as to which therapeutic regimen is optimal for treatment of LTBI specifically in children with CKD, on kidney replacement therapy or post kidney transplantation. There are a number of potential treatment regimens for LTBI, including isoniazid, rifampicin and rifapentine as monotherapy or combination treatment [55]. As noted above, drug regimen adjustments may be necessary during dialysis [54]. To avoid potential interactions of rifampicin with immunosuppressive medication post kidney transplant, isoniazid monotherapy appears the better option in that setting. The choice of regimen should therefore be discussed with a paediatrician with experience in the management of TB in children and a kidney and/or infectious diseases pharmacist.

#### TB disease

Guidance on the treatment of TB disease in children with CKD, kidney replacement therapy or post kidney transplantation is beyond the scope of this review. In general, treatment should follow standard national recommendations, giving additional consideration to drug interactions, kidney allograft function, potential drug toxicity and immune status. Treatment and monitoring should be guided by an expert in paediatric TB in close collaboration with the paediatric transplant team and a specialist pharmacist.

## Recommendations

In the light of the above review of evidence, the authors make the following recommendations relating to BCG and investigation and management of TB infection in countries with low TB incidence:

1. The indications for BCG immunisation in children with CKD are the same as in the general population. There should be close liaison with services providing BCG immunisation to ensure that children with CKD who meet the criteria for BCG immunisation receive their vaccination (if no other contraindications are present). In low TB prevalence countries, CKD without additional risk factors for TB exposure is not an indication for BCG immunisation. BCG immunisation is contraindicated in those receiving immunosuppression for kidney transplantation.
2. Due to their increased susceptibility to TB disease, all children with CKD should be referred promptly to TB services for clinical assessment if they develop any symptoms suggestive of possible TB disease. This is especially important in children aged less than 2 years old, after initiation of long-term kidney replacement therapy, and after immunosuppression for kidney transplantation.
3. All children with CKD should be screened by history for risk factors for potential TB exposure, and for known TB exposure. Any patient identified as having a history of contact or risk factor for exposure should have TST and IGRA performed as soon as possible.
4. If either TST or IGRA is positive, the child should be referred to a TB service for evaluation (including chest x-ray) to exclude TB disease; if there is no evidence of TB disease, the child should receive treatment for LTBI. Children who have indeterminate IGRA results and a negative TST should have a repeat IGRA. If the test result remains indeterminate, the patient should be referred for a chest x-ray and assessment by the TB team, who will decide whether LTBI treatment is warranted based on a risk assessment.
5. A history of new possible TB exposure, including extended travel to a high TB prevalence region, should be sought at each annual review.
6. At initiation of long-term kidney replacement therapy, and at decision to proceed to kidney transplantation, all children should have a further review of TB contact, of risk factors for TB exposure, and an IGRA and TST.
7. If a child on kidney replacement therapy or after immunosuppression for kidney transplantation has a significant new contact with a case of infectious TB, they should be re-assessed for TB disease. If there is no evidence of TB disease, they should be discussed with an expert in childhood TB for consideration for treatment for LTBI, irrespective of the TST and IGRA results considering the increased risk of false-negative results.
8. Children on kidney replacement therapy (but NOT children who are immunosuppressed for kidney transplantation) who have significant contact with respiratory TB and (a) have not been previously immunised with BCG, (b) have no evidence of TB disease and (c) remain negative by TST and IGRA at break-of-contact testing should be offered the BCG vaccine, in line with national immunisation guidelines (see Supplementary Tables 1–4, an example of national guidance and risk-based approach to BCG vaccination).
9. Treatment of LTBI and TB disease in children with CKD, on long-term kidney replacement therapy or after immunosuppression for kidney transplantation, should be supervised by an expert in the management of childhood TB in collaboration with the nephrology team and a specialist pharmacist. For fully sensitive TB, standard anti-TB regimens should be used (LTBI: isoniazid for six months or rifampicin plus isoniazid for three months; TB disease: rifampicin, isoniazid, pyrazinamide plus ethambutol for two months followed by rifampicin plus isoniazid for four months which may be extended for extrapulmonary/disseminated disease) and medication doses and frequency of administration should be tailored to the patient’s kidney function, kidney replacement therapy regimen and potential interactions with other medications. In addition therapeutic drug monitoring for anti-TB regimens should be considered.

## Supplementary Information

ESM 1 (DOCX 16 kb)

## References

[1] Becherucci F, Roperto RM, Materassi M, Romagnani P (2016). Chronic kidney disease in children. Clin Kidney J.

[2] Ploos van Amstel S, Noordzij M, Warady BA, Cano F, Craig JC, Groothoff JW, Ishikura K, Neu A, Safouh H, Xu H, Jager KJ, Schaefer F (2018). Renal replacement therapy for children throughout the world: the need for a global registry. Pediatr Nephrol.

[3] Plumb L, Wong E, Casula A, Braddon F, Lewis M, Marks SD, Shenoy M, Sinha MD, Maxwell H (2018). Chapter 4 demography of the UK paediatric renal replacement therapy population in 2016. Nephron.

[4] Lewis MA, Shaw J, Sinha MD, Adalat S, Hussain F, Castledine C, van Schalkwyk D, Inward C (2010). UK Renal Registry 12th Annual Report (December 2009): chapter 14: demography of the UK paediatric renal replacement therapy population in 2008. Nephron Clin Pract.

[5] Esmail H, Lai RP, Lesosky M, Wilkinson KA, Graham CM, Coussens AK, Oni T, Warwick JM, Said-Hartley Q, Koegelenberg CF, Walzl G, Flynn JL, Young DB, Barry Iii CE, O’Garra A, Wilkinson RJ (2016). Characterization of progressive HIV-associated tuberculosis using 2-deoxy-2-[(18)F]fluoro-D-glucose positron emission and computed tomography. Nat Med.

[6] Seddon JA, Jenkins HE, Liu L, Cohen T, Black RE, Vos T, Becerra MC, Graham SM, Sismanidis C, Dodd PJ (2015). Counting children with tuberculosis: why numbers matter. Int J Tuberc Lung Dis.

[7] Global tuberculosis report 2018. Geneva: World Health Organization; 2018. Licence: CC BY-NC-SA 3.0 IGO.

[8] England PH (2018). Tuberculosis in England: 2018.

[9] Houben RM, Dodd PJ (2016). The global burden of latent tuberculosis infection: a re-estimation using mathematical modelling. PLoS Med.

[10] British Thoracic Society Standards of Care Commission, Joint Tuberculosis Committee; Milburn H, Ashman N, Davies P, Doffman S, Drobniewski F, Khoo S, Ormerod P, Ostermann M, Snelson C (2010) Guidelines for the prevention and management of Mycobacterium tuberculosis infection and disease in adult patients with chronic kidney disease. Thorax 65:557–570. 10.1136/thx.2009.133173 10.1136/thx.2009.133173 20522863

[11] Ostermann M, Palchaudhuri P, Riding A, Begum P, Milburn HJ (2016). Incidence of tuberculosis is high in chronic kidney disease patients in South East England and drug resistance common. Ren Fail.

[12] Munoz P, Rodriguez C, Bouza E (2005). Mycobacterium tuberculosis infection in recipients of solid organ transplants. Clin Infect Dis.

[13] Bumbacea D, Arend SM, Eyuboglu F, Fishman JA, Goletti D, Ison MG, Jones CE, Kampmann B, Kotton CN, Lange C, Ljungman P, Milburn H, Morris MI, Muller E, Munoz P, Nellore A, Rieder HL, Sester U, Theodoropoulos N, Wagner D, Sester M (2012). The risk of tuberculosis in transplant candidates and recipients: a TBNET consensus statement. Eur Respir J.

[14] McCulloch MI, Gajjar P, Spearman CW, Burger H, Sinclair P, Savage L, Morrison C, Davies C, van Dugteren G, Maytham D, Wiggelinkhuizen J, Pascoe M, McCurdie F, Pontin A, Muller E, Numanoglu A, Millar AJ, Rode H, Khan D (2006). Overview of a paediatric renal transplant programme. S Afr Med J.

[15] Vecino R, Santiago B, Baquero-Artigao F, Lopez GL, Garcia C, Munoz G, Prieto G, Martinez A, de Jose MI, Mejias A (2012). Tuberculosis in pediatric solid organ and hematopoietic stem cell transplant recipients. Pediatr Infect Dis J.

[16] Newton SM, Brent AJ, Anderson S, Whittaker E, Kampmann B (2008). Paediatric tuberculosis. Lancet Infect Dis.

[17] Tebruegge M, Ritz N, Curtis N, Shingadia D (2015). Diagnostic tests for childhood tuberculosis: past imperfect, present tense and future perfect?. Pediatr Infect Dis J.

[18] Tebruegge M, Connell T, Ritz N, Orchard D, Curtis N (2010). Mycobacterium marinum infection following kayaking injury. Int J Infect Dis.

[19] Hermansen TS, Thomsen VO, Lillebaek T, Ravn P (2014). Non-tuberculous mycobacteria and the performance of interferon gamma release assays in Denmark. PLoS One.

[20] Gamsky TE, Lum T, Hung-Fan M, Green JA (2016). Cumulative false-positive quantiferon-tb interferon-gamma release assay results. Ann Am Thorac Soc.

[21] Pai M, Denkinger CM, Kik SV, Rangaka MX, Zwerling A, Oxlade O, Metcalfe JZ, Cattamanchi A, Dowdy DW, Dheda K, Banaei N (2014). Gamma interferon release assays for detection of Mycobacterium tuberculosis infection. Clin Microbiol Rev.

[22] Jarvis J, Gao Y, de Graaf H, Hughes S, Allan RN, Williams A, Marshall B, Elkington P, Faust SN, Tebruegge M (2015). Environmental temperature impacts on the performance of QuantiFERON-TB Gold In-Tube assays. J Inf Secur.

[23] Velasco-Arnaiz E, Soriano-Arandes A, Latorre I, Altet N, Dominguez J, Fortuny C, Monsonis M, Tebruegge M, Noguera-Julian A (2018). Performance of tuberculin skin tests and interferon-gamma release assays in children younger than 5 years. Pediatr Infect Dis J.

[24] Tebruegge M, de Graaf H, Sukhtankar P, Elkington P, Marshall B, Schuster H, Patel S, Faust SN (2014). Extremes of age are associated with indeterminate QuantiFERON-TB gold assay results. J Clin Microbiol.

[25] Meier NR, Volken T, Geiger M, Heininger U, Tebruegge M, Ritz N (2019). Risk Factors for indeterminate interferon-gamma release assay for the diagnosis of tuberculosis in children-a systematic review and meta-analysis. Front Pediatr.

[26] Luetkemeyer AF, Charlebois ED, Flores LL, Bangsberg DR, Deeks SG, Martin JN, Havlir DV (2007). Comparison of an interferon-gamma release assay with tuberculin skin testing in HIV-infected individuals. Am J Respir Crit Care Med.

[27] Cattamanchi A, Ssewenyana I, Davis JL, Huang L, Worodria W, den Boon S, Yoo S, Andama A, Hopewell PC, Cao H (2010). Role of interferon-gamma release assays in the diagnosis of pulmonary tuberculosis in patients with advanced HIV infection. BMC Infect Dis.

[28] Edathodu J, Varghese B, Alrajhi AA, Shoukri M, Nazmi A, Elgamal H, Aleid H, Alrabiah F, Ashraff A, Mahmoud I, Al-Hajoj S (2017). Diagnostic potential of interferon-gamma release assay to detect latent tuberculosis infection in kidney transplant recipients. Transpl Infect Dis.

[29] Lee SH, Kim HJ, Park SJ, Kim TH, Park SJ, Kang SW, Kim YH, Menzies D (2015). Serial interferon-gamma release assays for latent tuberculosis in dialysis patients with end stage renal disease in a Korean population. BMC Infect Dis.

[30] Rogerson TE, Chen S, Kok J, Hayen A, Craig JC, Sud K, Kable K, Webster AC (2013). Tests for latent tuberculosis in people with ESRD: a systematic review. Am J Kidney Dis.

[31] Triverio PA, Bridevaux PO, Roux-Lombard P, Niksic L, Rochat T, Martin PY, Saudan P, Janssens JP (2009). Interferon-gamma release assays versus tuberculin skin testing for detection of latent tuberculosis in chronic haemodialysis patients. Nephrol Dial Transplant.

[32] Southern J, Sridhar S, Tsou CY, Hopkins S, Collier S, Nikolayevskyy V, Lozewicz S, Lalvani A, Abubakar I, Lipman M (2019). Discordance in latent tuberculosis (TB) test results in patients with end-stage renal disease. Public Health.

[33] European Centre for Disease Prevention and Control. Use of interferon-gamma release assays in support of TB diagnosis. Stockholm: ECDC; 2011.

[34] Kato S, Chmielewski M, Honda H, Pecoits-Filho R, Matsuo S, Yuzawa Y, Tranaeus A, Stenvinkel P, Lindholm B (2008). Aspects of immune dysfunction in end-stage renal disease. Clin J Am Soc Nephrol.

[35] Kim SH, Lee SO, Park JB, Park IA, Park SJ, Yun SC, Jung JH, Kim YH, Kim SC, Choi SH, Jeong JY, Kim YS, Woo JH, Park SK, Park JS, Han DJ (2011). A prospective longitudinal study evaluating the usefulness of a T-cell-based assay for latent tuberculosis infection in kidney transplant recipients. Am J Transplant.

[36] Barton E, Gao Y, Ball D, Fidler K, Klein N, Curtis N, Clifford V, Marshall BG, Chancellor A, Mansour S, Elkington P, Tebruegge M (2019). Calcineurin inhibitors and variation in the performance of interferon-gamma release assays used to detect tuberculosis infection. Ann Am Thorac Soc.

[37] Clifford V, Zufferey C, Germano S, Ryan N, Leslie D, Street A, Denholm J, Tebruegge M, Curtis N (2015). The impact of anti-tuberculous antibiotics and corticosteroids on cytokine production in QuantiFERON-TB Gold In Tube assays. Tuberculosis (Edinb).

[38] Edwards A, Gao Y, Allan RN, Ball D, de Graaf H, Coelho T, Clifford V, Curtis N, Williams A, Faust SN, Mansour S, Marshall B, Elkington P, Tebruegge M (2017). Corticosteroids and infliximab impair the performance of interferon-gamma release assays used for diagnosis of latent tuberculosis. Thorax.

[39] Bakkaloglu SA, Ozdemir Atikel Y, Paglialonga F, Stefanidis CJ, Askiti V, Vidal E, Ariceta G, Melek E, Verrina E, Printza N, Vondrak K, Zurowska A, Zagozdzon I, Ekim M, Ozmert EN, Dufek S, Jankauskiene A, Schmitt CP, Levai E, Vande Walle J, Canpolat N, Holtta T, Fischbach M, Klaus G, Aufricht C, Shroff R, Edefonti A (2018). Vaccination practices in pediatric dialysis patients across europe. a European Pediatric Dialysis Working Group and European Society for Pediatric Nephrology Dialysis Working Group Study. Nephron.

[40] Danziger-Isakov L, Kumar D, AST ID Community of Practice (2019). Vaccination of solid organ transplant candidates and recipients: guidelines from the American society of transplantation infectious diseases community of practice. Clin Transpl.

[41] Miyairi I, Funaki T, Saitoh A (2016). Immunization practices in solid organ transplant recipients. Vaccine.

[42] Mangtani P, Abubakar I, Ariti C, Beynon R, Pimpin L, Fine PE, Rodrigues LC, Smith PG, Lipman M, Whiting PF, Sterne JA (2014). Protection by BCG vaccine against tuberculosis: a systematic review of randomized controlled trials. Clin Infect Dis.

[43] Roy A, Eisenhut M, Harris RJ, Rodrigues LC, Sridhar S, Habermann S, Snell L, Mangtani P, Adetifa I, Lalvani A, Abubakar I (2014). Effect of BCG vaccination against Mycobacterium tuberculosis infection in children: systematic review and meta-analysis. BMJ.

[44] Talbot EA, Perkins MD, Silva SF, Frothingham R (1997). Disseminated bacille Calmette-Guerin disease after vaccination: case report and review. Clin Infect Dis.

[45] Venkataraman A, Yusuff M, Liebeschuetz S, Riddell A, Prendergast AJ (2015). Management and outcome of Bacille Calmette-Guerin vaccine adverse reactions. Vaccine.

[46] Casanova JL, Jouanguy E, Lamhamedi S, Blanche S, Fischer A (1995). Immunological conditions of children with BCG disseminated infection. Lancet.

[47] Hesseling AC, Johnson LF, Jaspan H, Cotton MF, Whitelaw A, Schaaf HS, Fine PE, Eley BS, Marais BJ, Nuttall J, Beyers N, Godfrey-Faussett P (2009). Disseminated bacille Calmette-Guerin disease in HIV-infected South African infants. Bull World Health Organ.

[48] Bernatowska EA, Wolska-Kusnierz B, Pac M, Kurenko-Deptuch M, Zwolska Z, Casanova JL, Piatosa B, van Dongen J, Roszkowski K, Mikoluc B, Klaudel-Dreszler M, Liberek A (2007). Disseminated bacillus Calmette-Guerin infection and immunodeficiency. Emerg Infect Dis.

[49] Kinoshita N, Shoji K, Funaki T, Fukuda A, Sakamoto S, Kasahara M, Miyairi I (2018). Safety of BCG Vaccination in pediatric liver transplant recipients. Transplantation.

[50] Ziegler J, Ho J, Gibson IW, Nayak JG, Stein M, Walkty A, Orr P (2018). Disseminated Mycobacterium bovis infection post-kidney transplant following remote intravesical BCG therapy for bladder cancer. Transpl Infect Dis.

[51] Fox TG, Nailescu C (2019). Vaccinations in pediatric kidney transplant recipients. Pediatr Nephrol.

[52] Subramanian AK, Theodoropoulos NM (2019). Mycobacterium tuberculosis infections in solid organ transplantation: guidelines from the infectious diseases community of practice of the American Society of Transplantation. Clin Transpl.

[53] Ritz N, Tebruegge M, Connell TG, Sievers A, Robins-Browne R, Curtis N (2009). Susceptibility of Mycobacterium bovis BCG vaccine strains to antituberculous antibiotics. Antimicrob Agents Chemother.

[54] Saito N, Yoshii Y, Kaneko Y, Nakashima A, Horikiri T, Saito Z, Watanabe S, Kinoshita A, Saito K, Kuwano K (2019). Impact of renal function-based anti-tuberculosis drug dosage adjustment on efficacy and safety outcomes in pulmonary tuberculosis complicated with chronic kidney disease. BMC Infect Dis.

[55] Latent tuberculosis infection: updated and consolidated guidelines for programmatic management. Geneva: World Health Organization; 2018. Licence: CC BY-NC-SA 3.0 IGO 30277688

