# Supplementary material for: Preventing tuberculosis in paediatric kidney transplant recipients: is there a role for BCG immunisation pre-transplantation in low tuberculosis incidence countries?
Source: Pediatr Nephrol. 2020 Nov 27;36(10):3023–31. doi: 10.1007/s00467-020-04844-5 (PMC8445864; doi:10.1007/s00467-020-04844-5)
Supplement: Supplementary file 1 — (DOCX 16 kb) [file 467_2020_4844_MOESM1_ESM.docx]

**Supplementary Tables**

Supplementary Table 1: Indications for BCG immunisation in children in the UK (adapted from Green Book with kind permission of Public Health England [56, 57])

| BCG immunisation should be offered to:  ● all infants (aged 0 to 12 months) with a parent or grandparent who was born in a country where the annual incidence of TB is 40/100,000 or greater^†^  ● all infants (aged 0 to 12 months) living in areas of the UK where the annual incidence of TB is 40/100,000 or greater  ● previously unvaccinated children aged one to five years with a parent or grandparent who was born in a country where the annual incidence of TB is 40/100,000 or greater.^†^ These children should be identified at suitable opportunities, and can normally be vaccinated without TST testing  ● previously unvaccinated, TST-negative children aged from six to under 16 years of age with a parent or grandparent who was born in a country where the annual incidence of TB is 40/100,000 or greater.^†^ These children should be identified at suitable opportunities, TST tested and vaccinated if negative  ● previously unvaccinated TST-negative individuals under 16 years of age household or equivalent close contacts of cases of sputum smear-positive pulmonary or laryngeal TB (see National Institute for Health and Clinical Excellence (NICE), 2016)  ● previously unvaccinated, tuberculin-negative individuals under 16 years of age who were born in or who have lived for a prolonged period (at least three months) in a country with an annual TB incidence of 40/100,000 or greater.  ● those under 16 years who are travelling to stay with friends / family or local people for more than three months to a country where the annual incidence of TB is 40/100,000 or greater and/or where the risk of Multi Drug Resistant-TB (MDR-TB) is high^¥^ |
| --- |

† For country information on prevalence see: <https://www.gov.uk/government/publications/tuberculosis-tb-by-country-rates-per-100000-people>

¥ For country information with high rates of MDR-TB see: <http://www.who.int/tb/publications/global_report/en/>

Supplementary Table 2: Indications for TST testing before administration of BCG vaccine (adapted from Green Book with kind permission of Public Health England [56, 57])

| ● all individuals aged six years or over  ● infants and children under six years of age with a history of residence or prolonged stay (more than three months) in a country with an annual TB incidence of 40/100,000 or greater  ● those who have had close contact with a person with known TB  ● those who have a family history of TB within the last five years  BCG can be given up to three months following a negative TST (unless the patient had contact with a person with pulmonary TB in the interim). |
| --- |

Supplementary Table 3: Contraindications to BCG immunisation (adapted from Green Book with kind permission of Public Health England [56, 57])

| ● those who have already had a BCG immunisation in the past  ● those with a past history of TB  ● those with an induration of 5 mm or more following TST testing  ● those who have had a confirmed anaphylactic reaction to a component of the vaccine  ● children less than two years of age in a household where a TB disease case is suspected or confirmed  ● infants born to a mother who received immunosuppressive biological therapy during pregnancy  ● those who are receiving, or have received in the past 6 months:  -immunosuppressive chemotherapy or radiotherapy for malignant disease or non-malignant disorders  -immunosuppressive therapy for a solid organ transplant (with exceptions, depending upon the type of transplant and the immune status of the patient)  ● those who are receiving or have received in the past 12 months:  -immunosuppressive biological therapy unless otherwise directed by a specialist.  ● those who are receiving or have received in the past 3 months: immunosuppressive therapy including:  -children on high-dose corticosteroids (> 40 mg prednisolone per day above 20 kg, or > 2 mg/kg/day in children under 20 kg) for more than 1 week  - children on lower dose corticosteroids (> 20 mg prednisolone per day above 20 kg bodyweight, or > 1 mg/kg/day in children under 20 kg) for more than 14 days  -non-biological oral immune modulating drugs e.g. methotrexate > 25 mg per week, azathioprine > 3.0 mg/kg/day or 6-mercaptopurine > 1.5 mg/kg/day  ● BCG vaccine is absolutely contraindicated in all HIV-positive persons regardless of CD4 cell count, ART use, viral load, and clinical status  ● BCG should not be given to children with a primary immunodeficiency (e.g. SCID), unless this is deemed safe by a paediatric immunologist |
| --- |

Supplementary Table 4: Low dose immunosuppression which does not does not represent a contraindication to live vaccines, including BCG (adapted from Green Book with kind permission of Public Health England [56, 57])

| ● corticosteroids up to equivalent of 20 mg prednisolone per day for more than 14 days above 20 kg body weight, or 1 mg/kg/day in children under 20 kg either alone or in combination with other immunosuppressive drugs including:  ● low dose non-biological oral immune modulating drugs:  - methotrexate 25 mg per week above 20 kg or up to 15 mg/m^2^  - azathioprine 3.0 mg/kg/day  - 6-mercaptopurine 1.5 mg/kg/day |
| --- |
